# Text message intervention delivered from Australian general practices to improve breast cancer survivors’ physical activity and cardiovascular risk factors: protocol for the EMPOWER-SMS-GP effectiveness implementation randomised controlled trial

**DOI:** 10.1136/bmjopen-2024-090984

**Published:** 2024-12-11

**Authors:** Anna C Singleton, Stephanie R Partridge, Karice K Hyun, Christine Mitchell, Rebecca Raeside, Nashid Hafiz, Brooke Nickel, David Mizrahi, Allyson Ruth Todd, Jennifer McIntosh, Raymond J Chan, Kirsty E Stuart, Carolyn Ee, Elisabeth Elder, Julie Redfern

**Affiliations:** 1 The Daffodil Centre, a joint venture between the University of Sydney and Cancer Council New South Wales, The University of Sydney, Sydney, New South Wales, Australia; 2 Susan Wakil School of Nursing and Midwifery, The University of Sydney, Sydney, NSW, Australia; 3 Charles Perkins Centre, Faculty of Medicine and Health, The University of Sydney, Sydney, New South Wales, Australia; 4 ANZAC Research Institute, Concord Repatriation General Hospital, Concord, New South Wales, Australia; 5 School of Health Sciences, Faculty of Medicine and Health, The University of Sydney, Sydney, New South Wales, Australia; 6 Consumer Representative Group, Breast Cancer Network Australia, Camberwell, Victoria, Australia; 7 Sydney Health Literacy Lab, Sydney School of Public Health, Faculty of Medicine and Health, The University of Sydney, Sydney, New South Wales, Australia; 8 Wiser Healthcare, Sydney School of Public Health, Faculty of Medicine and Health, The University of Sydney, Sydney, New South Wales, Australia; 9 Melbourne School of Population and Global Health, The University of Melbourne, Melbourne, Victoria, Australia; 10 Caring Futures Institute, Flinders University, Adelaide, South Australia, Australia; 11 Westmead Breast Cancer Institute, Westmead Hospital, Western Sydney Local Health District, Sydney, New South Wales, Australia; 12 Radiation Oncology Network, Western Sydney Local Health District, Sydney, New South Wales, Australia; 13 Sydney Medical School, The University of Sydney, Sydney, New South Wales, Australia; 14 NICM Health Research Institute, Western Sydney University, Sydney, New South Wales, Australia; 15 Translational Health Research Institute, Western Sydney University, Sydney, New South Wales, Australia; 16 Institute for Evidence-Based Healthcare, Bond University, Gold Coast, Queensland, Australia

**Keywords:** Primary Care, Implementation Science, ONCOLOGY, Health Services, Cell Phone, PUBLIC HEALTH

## Abstract

**Introduction:**

Australian breast cancer survivors are at increased risk of cardiovascular disease and mortality, partly due to behavioural risk factors, including unhealthy diet and physical inactivity. Guidelines recommend health promotion delivered by general practitioners (GPs), but resources (ie, time and funding) are limited. Text message interventions sent from general practice to survivors may offer a low-resource solution but have not been evaluated. This randomised controlled trial (RCT) aims to evaluate the effectiveness and implementation of a text message intervention called EMPOWER-SMS-GP in Australian general practices.

**Methods and analysis:**

Multi-centre single-blind hybrid I RCT (n=678; 1:1 allocation) comparing EMPOWER-SMS-GP (n=339) to usual care (n=339) at 6 months (postintervention), 12, 18 and 24 months (maintenance) and parallel mixed-methods process evaluation using the Reach, Effectiveness, Adoption, Implementation and Maintenance framework. Inclusion: adults (≥18 years old) with early-stage breast cancer, completed active treatment ≤3 years ago, have a mobile phone and attended ≥1 GP appointment within 24 months. Primary outcome: between-group difference in mean physical activity (metabolic equivalent minutes/day) at 6 months, measured using an accelerometer. Secondary outcomes include self-reported physical activity, diet, quality of life, financial or psychological distress, fear of cancer recurrence, endocrine therapy adherence and body mass index. Statistical analyses (intention-to-treat) will include t-test (primary outcome) and linear and logistic mixed-effects regression models.

**Ethics and dissemination:**

Approval received from the University of Sydney Human Research Ethics Committee (Number 2023/081). Trial results will be disseminated in peer-reviewed publications, presentations, lay summaries, videos and audio for scientific, government and public audiences.

**Trial registration number:**

Australia and New Zealand Clinical Trial Registry (ACTRN12624000591550, 09/05/2024; U1111-1307-3454).

**STRENGTHS AND LIMITATIONS OF THIS STUDY**

- This is a high-quality evaluation of the short-term (6 months) and long-term (12, 18, 24 months) effectiveness of low-cost, scalable, co-designed text message intervention sent from general practice to manage survivors’ risk factors compared with usual care.
- Evaluating barriers and enablers to implementation into general practices using the Reach Effectiveness Adoption Implementation and Maintenance framework will provide important insights into practice staff’s and patients’ perspectives.
- Recruiting from general practices across Australia will improve the likelihood of a representative sample of participants.
- Enrolment and electronic consent via the Internet can enable rapid recruitment of participants. However, it may also cause limitations for individuals with lower digital health literacy.
- Measuring physical activity (primary outcome) with wrist-worn accelerometers will provide objective data with high sensitivity. However, non-adherence may lead to missing data.

## Introduction

 Breast cancer is one of the most commonly diagnosed cancers in Australia. Five-year survival rates for people with early-stage breast cancer are high (92%, 2015–2019), representing over 81 383 people who require long-term medical care.^1^ Breast cancer survivors are at increased risk of cardiovascular disease (CVD) and cancer recurrence, in part, due to overlapping risk factors, including eating an unhealthy diet (eg, insufficient vegetables and fruit) and physical inactivity.^2^ An Australia-based randomised controlled trial (RCT; n=160) and national survey (n=309) found that only 11% of breast cancer survivors met Australian guidelines for vegetable intake (≥5 servings per day), 38% for fruit intake (≥2 servings per day) and 37% for physical activity (150 min of moderate or 75 min of vigorous physical activity per week).^3 4^ Improving adherence to risk factor guidelines, especially physical activity, through education and structured programmes can improve health outcomes, including weight, anxiety and quality of life (QoL).^3 5^ Furthermore, recent meta-analyses and large-scale prospective studies found that even small improvements in physical activity (75 min of moderate or 15–20 min of vigorous physical activity per week) can significantly reduce risks of CVD, breast cancer-specific mortality and all-cause mortality.^6 7^ However, patients face significant personal and systemic barriers to accessing physical activity and other health-promoting services, including limited finances, inconvenient programme location and/or time.^3^ Therefore, improving access to health-promoting services for breast cancer survivors through novel multidisciplinary strategies has become a priority for national peak bodies, including the Clinical Oncology Society of Australia and the Primary Care Collaborative Cancer Clinical Trials Group, with general practice playing a central role.^8 9^

General practitioners (GPs) are well-positioned to support breast cancer survivors’ long-term health outcomes. In 2019, Cancer Australia established shared-care guidelines for specialists and GPs to jointly deliver breast cancer survivors’ long-term follow-ups, including delivery of health education about risk factors and referrals to relevant programmes. This model of care was found safe, effective, cost-effective and acceptable to GPs and patients.^10^ In the same year, the Royal Australian College of General Practice’s national physical activity and nutrition counselling survey (n=657) found that 80% of GPs and registrars felt that providing physical activity and nutrition counselling was core to their role with 87% reporting they provide this.^11^ In a survey of Australian GPs who regularly see patients living with cancer (n=111), most GPs perceived physical activity to be safe (90%) and beneficial (91%) for patients, but only recommended physical activity to 41–60% of their patients and referred 1–20% of their patients to physical activity programs.^12^ Australian GPs face many barriers to delivering risk factor education or referrals, including lack of adequate funding for longer preventive health appointments and limited time during appointments, especially for patients with multi-morbidities.^11 12^ Inexpensive and time-saving strategies to support GPs in their goals to deliver risk factor advice to breast cancer survivors are needed.^13^

General practice text message reminder systems may be a low-resource (time, cost) solution to supporting GPs in delivering risk factor education and programme referrals to breast cancer survivors between appointments. Text messaging interventions that deliver evidence-based education about risk factors and weblinks to resources to people with chronic diseases have been found effective in improving health outcomes, including physical activity levels, healthy eating, smoking cessation and medication adherence.^14^ In Australia, our team conducted a pilot RCT (n=160) in an oncology clinic that found a co-designed text message intervention called EMPOWER-SMS effective in improving endocrine therapy medication adherence compared with control (usual care, no text messages).^4^ Although the pilot was underpowered for other outcomes, results trended towards the EMPOWER-SMS group having a greater proportion of participants than the control group that met Australian vegetable intake guidelines (15% vs 12%) and higher mean physical activity levels (187 vs 164.8 metabolic equivalents (MET) minutes/day), which equates to a clinically meaningful difference (~5.5 min/day) for reducing risks of CVD and cancer.^7^ Furthermore, EMPOWER-SMS was tested in a community-based implementation pilot and was found to have a high uptake (n=841) among socioeconomically diverse breast cancer survivors, with 31.5% residing in regional, rural and remote areas or 30% in low socioeconomic areas.^15^ Direct costs to send EMPOWER-SMS were low (~AUD$11/participant/6 months) and required little staff oversight (75 min/week/841 participants). Qualitative feedback from these two studies (n=513) revealed that most participants ‘strongly agreed’ or ‘agreed’ that EMPOWER-SMS was useful (91%), supportive (88%) and motivating for improving physical activity (70%) and healthy eating (69%) and participants wanted to receive EMPOWER-SMS from their GP.^4 15^ This aligns with another Australian trial (‘SMARTscreen’) that found that sending a text message from general practice that reminded patients about bowel cancer screening increased uptake of bowel cancer screening compared with usual care.^16^ However, the delivery of a text message-based risk factor management programme sent from general practice to improve breast cancer survivors’ health outcomes has yet to be evaluated.

This multi-centre hybrid one effectiveness-implementation RCT will provide new robust, objective evidence for the short-term (6 months) and long-term (12, 18 and 24 months) effectiveness of an adapted version of EMPOWER-SMS called ‘EMPOWER-SMS-GP’ sent from general practice compared with usual care, and barriers and enablers to implementation. Specifically, the trial aims to evaluate (1) the effectiveness of EMPOWER-SMS-GP on improving physical activity measured using an accelerometer (mean MET-minutes/day; primary outcome) and secondary outcomes, including changes in diet, QoL, distress, financial distress, fear of cancer recurrence, endocrine therapy medication adherence and body mass index (BMI) at 6 months (postintervention); (2) the maintenance of outcomes at 12, 18 and 24 months, and (3) barriers and enablers to implementation into general practice from patients’ and practice staff’s perspectives using the Reach Effectiveness Adoption Implementation and Maintenance (RE-AIM) framework.^17^ It is hypothesised that EMPOWER-SMS-GP will be effective in improving breast cancer survivors’ physical activity levels and secondary outcomes and will help inform future implementation and scale-up of risk factor management text message programmes into general practice for breast cancer survivors analysis across Australia.

## Methods and analysis

### Trial design

**Figure 1:**
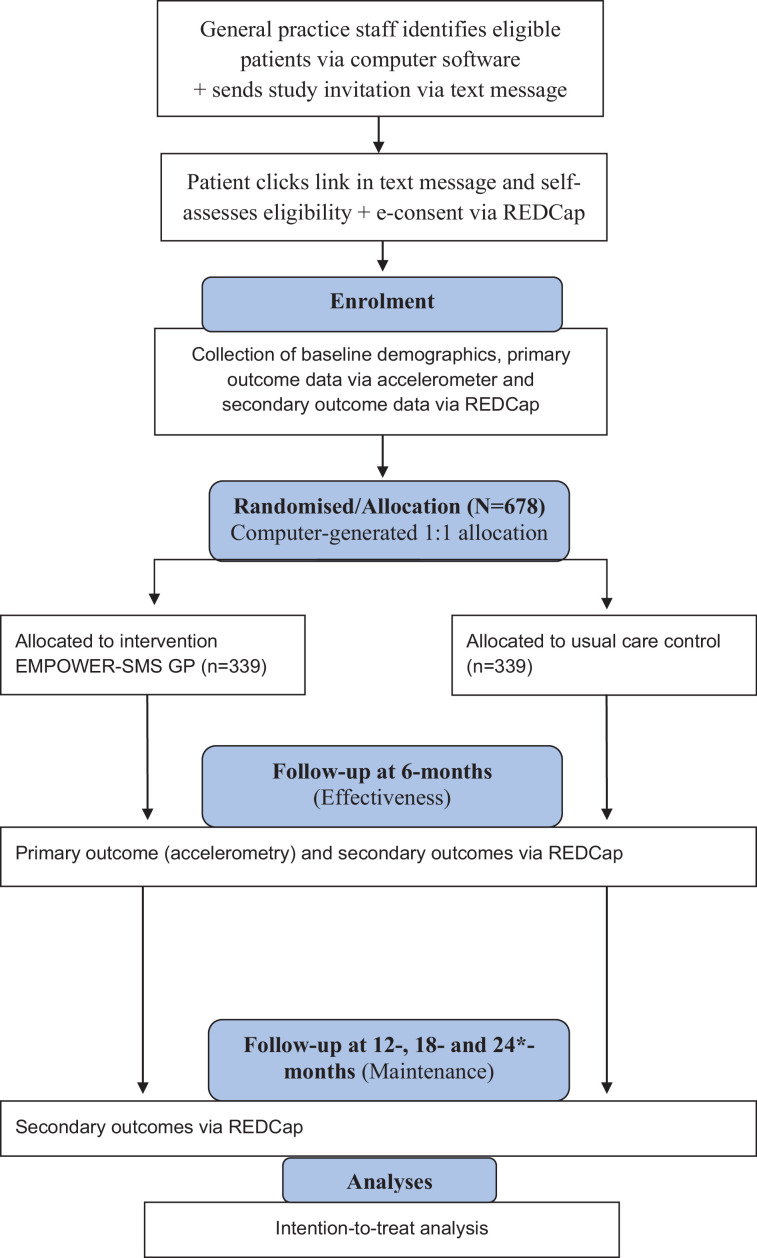
Consolidated Standards of Reporting Trials flow diagram. *If funding and time allow. RE-AIM, Reach Effectiveness Adoption Implementation and Maintenance, REDCap, Research Electronic Data Capture.

A multi-centre single-blind hybrid I RCT (n=678; 1:1 allocation ratio) evaluating the effectiveness of EMPOWER-SMS-GP (intervention; n=339) on participants’ health outcomes compared with usual care alone (control; n=339) at 6 months (postintervention), 12, 18 and 24 months* (maintenance; figure 1), and implementation through a parallel process evaluation using the enhanced RE-AIM Framework.^17^ The protocol was prepared following the Standard Protocol Items: Recommendations for Interventional Trials guidelines and Patient-Reported Outcomes extension (online supplemental appendix 1). The trial sponsor is the University of Sydney, Australia.

### Patient and public involvement in research

Patients and consumer representatives have been involved in this research as active steering committee members and/or coauthors from the original EMPOWER-SMS trial to the current trial. Patients and consumer representatives co-designed the EMPOWER-SMS intervention with health professionals and researchers, including choosing the length of the programme (6 months), the message content and the number and timing of messages received each week.^18^ Based on feedback from over 500 Australian breast cancer survivors,^4 15^ the intervention was adapted for general practice (‘EMPOWER-SMS-GP’). For the current trial, consumer representatives provided important advice regarding the study design and selection of outcomes, especially the feasibility and acceptability of wrist-worn accelerometers, the length of questionnaires and recruitment materials. They also informed dissemination plans, including infographics, videos and lay summaries.

### Eligibility criteria and recruitment

The inclusion criteria are: (1) adults (aged 18 years or older), (2) any gender, (3) diagnosed with early-stage (0-III) breast cancer, (4) completed active treatment including surgery and/or chemotherapy and/or radiation therapy within the past 3 years, (5) own a mobile phone and (6) attended at least one appointment at a participating general practice within the past 24 months. Participants will be excluded if they have an unsuitable diagnosis, for example, distant metastatic breast cancer, are already participating in a text message or risk factor management research study or are unable to comply with study requirements, including wearing an accelerometer or safely participating in physical activity or eating vegetables (not limited for medical reasons).

Participants will be recruited from at least 20 Australian general practices. Practices can be located in any state or territory and will be purposively sampled for postcode, practice type (corporate vs non-corporate) and practice size. Practices must have software that enables the identification of eligible patients and the delivery of text messages. General practices will send an initial invitation text message to eligible patients with brief information about the study and a weblink to the Research Electronic Data Capture (REDCap) website, which includes the patient information sheet and electronic consent. The research team’s contact details will be available to answer questions if needed. If recruitment is stagnated due to current general practice staff capacity challenges, then convenience sampling may be used. Study procedures will be pilot-tested with a small number of practices (n=2–5) to identify and promptly resolve recruitment issues.

### Group allocation

The intervention group (EMPOWER-SMS-GP) will receive usual care, plus a text message support programme that delivers four to five text messages per week for 6 months (104 messages total) regarding health education and self-management for physical activity, healthy diet, medication adherence, managing side effects, mental health and weblinks to free health resources. The EMPOWER-SMS-GP message content was adapted from the co-designed EMPOWER-SMS programme based on participant feedback (n>500) and recent research and general practice guidelines for health promotion for cancer survivors.^4 6 8 9 15 18^ Messages are positively toned, semi-personalised with the participant’s preferred name and designed to be appropriate for individuals with a grade 7 (Flesch Kincaid) reading level.^18^ Participants can reply, but it is not mandatory, and can opt out by replying ‘STOP’. An unblinded clinical research team member will monitor replies for safety and respond if needed including requests to withdraw.

The control group will receive usual care alone from their assigned health professionals, plus a text message with their group allocation. After the final follow-up at 24 months*, control group participants will be offered the EMPOWER-SMS-GP intervention for free. This will not be evaluated.

### Outcomes and data collection

The primary outcome is the between-group (intervention vs control) difference in physical activity (mean MET-min/day) at 6 months, using GENEActiv accelerometers. GENEActiv is a wrist-worn accelerometer that collects continuous data and has been validated for continuous patient monitoring. Participants only wear the accelerometer for 7 days at baseline and 6 months follow-up. The accelerometer does not provide feedback to the wearer (no screen), minimising the likelihood of participants’ physical activity being impacted by accelerometer information. Accelerometers will be sent to and from participants in a reply-paid envelope, with wear instructions.^19^ Secondary outcomes, including physical activity, diet, QoL, distress, financial distress, fear of cancer recurrence, endocrine therapy medication adherence and BMI, will be collected at baseline, 6, 12, 18 and 24 months* (*if capacity/funding allow; figure 1; table 1) using accelerometers or English versions of validated or previously used self-reported questionnaires^420^,^27^ on REDCap or over the phone, in the same order every time. To reduce avoidable missing data, participants will be sent reminders (email, text message), and REDCap data quality features will be enabled. Linkage and automated extraction of de-identified clinical data from general practice, including diagnosis, medication lists and weight, will occur at baseline, 6 and 18 months.

**Table 1:** Schedule of study procedures

Schedule of assessments	Study period					
	Baseline/enrolment	Allocation	Effectiveness	Maintenance		
TIME POINT	*-t_1_*	*t*_0_	*t_1_* 6 months±6 weeks	*t_2_* 12 months±6 weeks	*t_3_* 18 months±6 weeks	*t_4_* 24 months±6 weeks
**ENROLMENT**						
Eligibility screen	✓					
Informed consent	✓					
Randomisation/allocation		✓				
**INTERVENTION**						
EMPOWER-SMS-GP text message programme		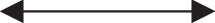				
**ASSESSMENTS**						
**Primary outcome**						
Physical activity (mean MET-min/day), measured using accelerometers^19^	✓		✓			
**Secondary outcomes**						
Physical activity, proportion (%) of participants meeting risk-factor reduction targets (≥600 MET-min/week; ≥300 MET-min/week), measured using accelerometers^19^	✓		✓			
Physical activity and vegetable intake, proportion meeting risk-factor reduction targets (≥600 MET-min/week AND≥5 servings of vegetables/day), measured using accelerometers and self-report questions from Sax Institute’s 45&Up study^19 21^	✓		✓			
Physical activity, mean per week and proportion (%) of participants meeting risk-factor reduction target (Leisure-time Index Score≥24), measured using the Godin-Shephard Leisure-Time Physical Activity Questionnaire^20^	✓		✓	✓	✓	✓
Diet, proportion of participants meeting risk-reduction targets (≥5 vegetable servings/day, ≥3 vegetable servings/day, ≥2 fruit servings/day, ≥2 fish servings/week, ≤7 red meat servings/week, ≤7 (women) or ≤14 (men) standard alcoholic drinks per week), measured using self-report questions from Sax Institute’s 45&Up study^21^	✓		✓	✓	✓	✓
Quality of life (mean), measured using the EORTC QLQ-C30* QoL/Global Health Status subscale^22^ and EQ5D†^23^	✓		✓	✓	✓	✓
Distress (mean; score<4), measured using the National Comprehensive Cancer Network’s Distress Thermometer^24^	✓		✓	✓	✓	✓
Financial distress (mean), measured using the National Medical Center and Beckman Research Institute QoL Instrument Breast Cancer Patient Version^25^	✓		✓	✓	✓	✓
Fear of cancer recurrence (FCR; mean; score<5), measured using the FCR-1r^26^	✓		✓	✓	✓	✓
Endocrine therapy medication adherence‡, proportion of participants missing <1 tablet per week, measured using fit-for-purpose self-report questionnaire^4^	✓		✓	✓	✓	✓
Body mass index, mean (kg/m^2^) and proportion (%) meeting risk-reduction target (18.5–24.9 kg/m^2^), measured using self-reported height (m) and weight (kg)^27^	✓		✓	✓	✓	✓
Cancer recurrence rate, proportion (%) of participants who report a recurrence, measured using a self-reported fit-for-purpose yes/no question			✓	✓	✓	✓
**Process evaluation**						
User feedback survey§^4^			✓			
Focus groups§			✓			
General practice staff feedback survey			✓			
General practice staff semi-structured interviews			✓			
Text message delivery and reply data^4^		✓	✓			

* European Organisation for Research and Treatment of Cancer Quality of Life Questionnaire.

† EuroQol-5 Dimension.

‡ Only for participants taking endocrine therapy medication.

§ Only for participants in the intervention group.

GPgeneral practitionerMETmetabolic equivalent

### Sample size

Based on physical activity levels among breast cancer survivors in our pilot study,^4^ a sample size of 678 (339/group) will achieve 80% power to detect a 22.2 difference in mean physical activity MET-minutes/day between the intervention group (187.0 MET-min/day) and control group (164.8 MET-min/day) with population SD of 91.96 after accounting for 20% dropout. The significance level of the two-sided tests used was 0.05.

### Randomisation, blinding and concealment

The randomisation sequence will be managed by an independent statistician and generated using a computer programme (SAS, V.9.4; 1:1 blinded allocation, random permuted block sizes of 4 and 6). Study groups will be concealed using pseudonyms, ‘group A’ and ‘group B’. After participants complete baseline enrolment procedures, study personnel will randomise participants to ‘group A’ or ‘group B’ (1:1 allocation ratio) using a secured computerised randomisation programme in REDCap, which triggers delivery of a text message to participants disclosing their group allocation (EMPOWER-SMS-GP intervention or control). Therefore, study personnel managing data collection and the statistician conducting outcome assessments will be blinded to group allocations.

### Statistical analyses

Statistical analyses will follow intention-to-treat and a prespecified Statistical Analysis Plan led by our blinded statistician, using SAS, V.9.4 and tests will be two-tailed. Categorical and continuous data will be summarised as frequencies and percentages or means and SD, respectively. For the primary outcome, a t-test will be used. As a sensitivity analysis, a linear mixed-effects regression model will be used to account for the practice clustering effect and baseline values of the primary outcome. Pre-specified sub-analysis will include major cities versus regional/rural/remote, age, gender, health literacy level, socioeconomic status and reported engagement (high vs low engagement determined by user survey). In case of non-negligible amounts of missing data (>10%), multiple imputations will be used under the missing at random assumption. For the secondary continuous and categorical outcomes, linear and logistic mixed effects regression models will be used, respectively, to account for the practice clustering effect and adjust for the outcome values at baseline.

### Process evaluation

A mixed-methods approach will be used following the enhanced RE-AIM framework (table 2).^17^ Setting-level and patient-level data sources will include participant demographics, text message delivery data, and patient and general practice staff feedback surveys. In addition, focus groups will be conducted with intervention participants and semi-structured interviews will be conducted with general practice staff, including GPs, practice managers, practice nurses and receptionists. The sample size will be based on thematic saturation. However, it is estimated that approximately 24 intervention participants and 24 practice staff members will be required. Maximum variation sampling will be used based on participants’ postcode, age and ancestry to ensure a range of views are obtained. The focus groups and interviews will be conducted via videoconference or phone by a trained interviewer using a discussion guide and will be audio recorded and transcribed verbatim. Transcripts will be de-identified and provided to participants for review on request. Quantitative data will be summarised using frequencies and percentages or means and SD. Qualitative data will be parallel coded by two researchers with qualitative research experience in NVivo (QSR International) using the enhanced RE-AIM framework.^17^ Codes will be compared for inter-coder reliability and disagreements will be discussed with a third independent researcher until an agreement is reached. Direct quotations will be used as exemplars of overarching themes and subthemes.

**Table 2:** Process evaluation using the enhanced RE-AIM framework

Framework category	Measurements
Reach	Proportion of eligible patients enrolled (n enrolled/n eligible × 100)
Representativeness	Demographics: age (years), ancestry, country of birth, gender identity, work status, treatment type, time since completing active treatment. Postcode will be used to determine participants’ Index of Relative Socioeconomic Advantage and Disadvantage (quintiles from one most- to five least disadvantaged area) and Australian Statistical Geography Standard of Remoteness Areas (categorise: Major City, Inner Regional, Outer Regional, Remote, Very Remote)
Effectiveness	Effect size of primary and secondary outcome (95% CI)
Adoption	Number, proportion and representativeness of participating practices, based on the practice postcode and size (number of patients per year); and reasons for non-adoption (declining to deliver the study)
Implementation	
Patient-reported experience measures (acceptability, utility, engagement)	User feedback survey (intervention group only): 17 questions, 15 multiple-choice with 5-point Likert scale (1=strongly disagree to 5=strongly agree), yes/no or select all that apply and two free-text responsesFocus groups with a subset of intervention participants (approximately 24 participants)Text message delivery data: number, and content of text message replies, including text, emojis (a small digital image or icon used to express an idea or emotion) or ‘reactions’, which occur when a participant clicks a text message and clicks ‘like’, ‘love’ or ‘emphasise’
Intervention Fidelity	Text message delivery data: number of text messages successfully delivered or unsuccessfully delivered (bounced). Percentage of opt-outs (n opt-outs/n baseline × 100), based on ‘STOP’ or opt-out request text message replies from intervention participantsAdaptations to the intervention or implementation processes: Log of any changes made during the study period
Feasibility	Setting-level direct intervention costs, including delivering the text messages, monthly fees for the automated text message delivery software, including general practice computer software.Individual-levelFit-for-purpose feedback survey for general practice staff members (GPs, allied health, practice managers or receptionists) to identify barriers/enablers to implementation, estimated general practice staff time and costs to identify eligible patients and send text message invitation. Semi-structured interviews with staff members (GPs, allied health, practice managers, receptionists)
Maintenance	Between-group differences in secondary outcomes (effect size, 95% CI) at 12, 18 and 24 months*

* if capacity/funding allow.

GPgeneral practitionerREDCapResearch Electronic Data Capture

### Ethics statement

This study will be conducted in full conformance with principles of the ‘Declaration of Helsinki’, Good Clinical Practice, the National Health and Medical Research Council(NHMRC) of Australia’s National Statement on Ethical Conduct in Human Research (2007) and the conditions of the University of Sydney Human Research Ethics Committee (HREC Approval number 2023/081; Protocol V.3), including safety monitoring, auditing and reporting of adverse events. All participants will provide informed e-consent (online supplemental appendix 2). Any protocol amendments will be reported as required to relevant parties, including the HREC.

### Dissemination of findings

De-identified or group-level trial results, including qualitative data, will be published in peer-reviewed scientific journals and formal reports to organisations, including the World Cancer Research Fund, Breast Cancer Network Australia, and presented at national and international conferences. The results will also be promoted in the media (television/radio/print/social), public newsletters and events that advocate for improved health, including breast cancer luncheons and fundraising events.

## supplementary material

10.1136/bmjopen-2024-090984 online supplemental file 1

10.1136/bmjopen-2024-090984 online supplemental file 2
