# Supplementary material for: Text message intervention delivered from Australian general practices to improve breast cancer survivors’ physical activity and cardiovascular risk factors: protocol for the EMPOWER-SMS-GP effectiveness implementation randomised controlled trial
Source: BMJ Open. 2024 Dec 11;14(12):e090984. doi: 10.1136/bmjopen-2024-090984 (PMC11647304; doi:10.1136/bmjopen-2024-090984)
Supplement: online supplemental file 2 [file bmjopen-14-12-s002.docx]

**What is this study about?**

This research study is testing if a new text message program called EMPOWER-SMS GP can help people stay healthy after breast cancer treatment compared to not getting any text messages.

This Participant Information Statement tells you about the research study. Knowing what is involved will help you decide if you want to take part. Please read this sheet carefully and ask questions about anything that you don’t understand or want to know more about. Taking part in this research study is voluntary. By giving your consent to take part in this study you are telling us that you:

- Understand what you have read.
- Agree to take part in the research study as outlined below.
- Agree to the use of your personal information as described.

Your decision to take part, or not, will not affect your current or future relationship with your healthcare provider, the researchers or anyone else at The University of Sydney.

**Who is running the study?**

The study is being carried out run by the following researchers from the University of Sydney (Professor Julie Redfern, Dr Anna Singleton, Dr Stephanie Partridge, Associate Professor Elisabeth Elder, Dr Kirsty Stuart, Dr Karice Hyun, Ms Allyson Todd, Ms Nashid Hafiz), Western Sydney University (Associate Professor Carolyn Ee), Flinders University (Dr Raymond Chan) and consumer representative Ms Molly Regimbal. This study is being funded by a World Cancer Research Fund International Grant and a National Health Medical Research Institute Investigator Grant.

**Who can take part in the study?**

To be eligible for this study, you will be:

- An adult of any gender (aged 18 years or older) who owns a mobile phone
- Completed active treatment (surgery, chemotherapy and/or radiation therapy) for early-stage (0-3) breast cancer within the past 3 years. Please note, you can still be on targeted therapy (e.g. Herceptin) or taking endocrine (anti-hormone) therapy tablets (e.g. Tamoxifen or aromatase inhibitors).
- **Willing to wear an activity watch for 7 days**
- Able to read and understand English at a 7^th^ grade level
- Able to safely do all study activities, like physical activities of your choice and eat vegetables (not limited for medical reasons)

Because the study was designed by, and for, people with early-stage breast cancer, you are not able to take part if you are (i) diagnosed with distant metastatic breast cancer, (ii) still receiving active breast cancer treatment or (iii) already taking part in a text message-based study or clinical trial. This is because the program may not be suitable for people who may need more personalized support.

**What will the study involve for me?**

1. **Fill in online surveys** today and then again in 6-, 12-, 18- and 24-months. The surveys take 5-10 minutes to fill in, depending on the month. The questions are about you and your health, like your name, date of birth, gender, mobile phone number and time since finishing active breast cancer treatment. The surveys will be easy to access via a link in a text-message or email, but can be done over the phone, if you want.


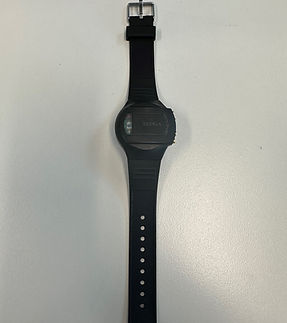

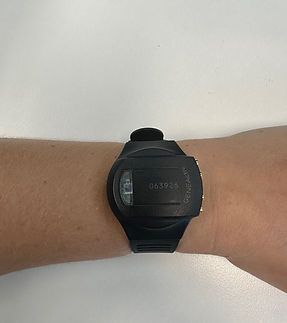
2. **Wear an activity watch for 7 days** at the start of the study (this week) and again 6-months later. This is so we can check for any changes in your physical activity levels. The activity watch is water and impact resistant (see image) and only collects data on physical activity and sleep. It cannot track your location. You will receive the activity watch in the mail with instructions and a pre-paid postage bag to return it to the research team.

3. You will have a **50% chance of being in put in one of these groups**:
**- EMPOWER-SMS-GP (‘Intervention’) group** will receive text messages about general health education, simple ways simple ways to get active, manage side-effects, eat healthy and stay well. Some messages will include links to helpful websites and free health programs. The text messages are written in simple English, around 1-2 sentences each. Messages will be semi-personalised with your preferred name and if you are taking endocrine therapy. You can expect 4 to 5 text messages from us each week for 6-months. You can read the message at a time that suits you and you can reply if you wish, but it is not required. You can stop the messages at any time by replying ‘STOP’. A research team member will see all replies and respond, if necessary, within 72 hours during office hours.

**- Standard Care (‘Control’) Group** will receive usual care for 6-months. You will be offered the EMPOWER-SMS-GP program free-of-charge at the end of the study.

All the text messages will be sent through a web-based text message platform called Burst SMS (ABN 40 116 431 700). Burst SMS abides by Australian Data Protection Laws and has entered a legal agreement with the University of Sydney for this study.

The groups are chosen at random by a computer system. It is not possible for you to choose the group, nor will you be able to change groups at any time. The researchers and primary care staff will also have no control over what group you are in. You will be told which group you are in via a text-message once you have signed up for the study.

Our study is a ‘single-blinded randomised controlled trial’. This means the researchers will not know which group you are in, and please do not tell them. This is to make sure the researchers interpret the results in a fair way and limits bias. At the end of the study, the results will tell us if the EMPOWER-SMS GP program improved breast cancer survivors’ health compared to standard care, or not.

**Focus group (optional)**: If you received the EMPOWER-SMS-GP program, you will be invited via a text-message, email or phone call to take part in a focus group, which is a group discussion with researchers and other participants to share your feedback about the program. You will be asked questions about if the program was useful, acceptable and how to improve it. It will take around 1-hour and will be online via a teleconference (e.g., Zoom). If you take part, you will be repaid with a $50 gift voucher to thank you for your time. This payment is suggested by the Health Consumers NSW guidelines.

You will have the option to turn your cameras off during the Zoom meeting. The meeting will be audio-recorded, and all participants’ names and identities will be removed (‘de-identified’) from a written transcript that will be used for data analysis purposes only. You can get a copy of the transcript upon request. Taking part in the focus group is completely voluntary. You may choose to withdraw at any time, but any information collected until that time cannot be removed from the group audio recording.

**What if I need medical advice?**

The Study does not and cannot replace the care, advice and help from qualified healthcare professionals. If you have any questions about your health (including medical condition and/or nutrition), please contact a qualified healthcare professional (e.g., your general practitioner or accredited practicing dietician). Also, the Study should never be used for medical emergencies. In life threatening or emergency situations, please call Triple Zero (000) for Police, Fire or Ambulance.

**Can I stop taking part (withdraw) once I’ve started?**

Yes, you can. If you decide to take part in the study and then change your mind, you can withdraw at any time. You can do this by replying ‘STOP’ to any of the messages and one of the research team will stop the messages as soon as they can. If you decide to withdraw, we will not collect any more information from you. Any personal information already collected will be retained to ensure that the results of the research study can be measured properly and comply with the law. Any information that we have already collected prior to withdrawal may be included in the study results.

**Are there any risks or costs?**

Taking part in this study is free-of-charge*.* You are responsible for the payment of personal fees with your mobile phone carrier, including monthly subscription fees, fees for any messages that you send to us, and any internet data usage from weblinks sent you. In Australia, the majority of mobile phone subscription plans do not charge their customers for receiving standard in-coming text messages. If you are unsure, please clarify with your provider before signing up for the study.

For safety purposes, we encourage participants to only read and respond to text messages at acceptable times and must not use their mobile phone while driving.

Aside from giving up your time, we do not expect that there will be any significant risks associated with you taking part in this study. There is a very low risk that another person could access health information via your mobile phone. This can be minimised by ensuring your phone or hand-held device has a secure passcode lock and message preview function is disabled. If you are feeling distressed or upset anytime throughout the study, here are services that you can talk to and websites you can access:

Lifeline T: 13 11 14 W: lifeline.org.au

Beyond Blue T: 1300 22 4636 W: beyondblue.org.au

Cancer Council T: 13 11 12 W: cancercouncil.com.au/get-support/

**Are there any benefits?**

We hope that participants will benefit from the information they receive though the text messages. In our past studies, feedback from over 500 breast cancer survivors found that EMPOWER-SMS GP was easy-to-understand (99%), useful (91%), motivating for exercise and healthy eating (70%) and helped people feel supported (87%) between medical appointments. It is possible that taking part in this study may help you manage your health, but it is not guaranteed. There may also be benefits to other people because if the messages are useful then they may be made available to more people.

**What will happen to information that is collected?**

By giving your consent, you are agreeing to us collecting, storing, and using personal information about you for the purposes of this research study. Only authorised staff members will have access to this information. All information will be treated confidentially and stored securely on the University of Sydney research portal (shared drive) for 15 years after completion of the study then permanently deleted, accordingly to ethical, legal and government rules. Survey responses will be collected via a University of Sydney-approved secure website called REDCap. To receive the text messages, your first name, mobile number and any replies that you send us will also be securely stored on the Burst SMS according to their privacy policy (https://burstsms.com/legal/privacy-policy). Only researchers on this study have password-protected access to the Burst SMS account. We are also working with your primary care practice which uses computer software that allows the auto-extraction of health data. By giving us your personal information (for example Medicare number [optional] and birthdate), you are agreeing to the extraction of data from your GP (for example medications and medical history).

Any information about you will only be disclosed with your permission, unless we are required by law. We anticipate study findings about the groups will be published. You will not be individually identifiable in these publications.

**What happens when the study ends? Will I be told the results of the study?**

Yes, you have a right to receive feedback about the overall results of this study. We will give all participants with a short summary of the results (around 1 A4 page) via your email address.

**What if I would like further information?**

The following researcher/s are available to discuss the study with you and answer any questions you may have: Dr Nashid Hafiz University of Sydney Ph: [insert study mobile], Email: [nashid.hafiz@sydney.edu.au](mailto:nashid.hafiz@sydney.edu.au) or Dr Anna Singleton, Email: [anna.singleton@sydney.edu.au](mailto:anna.singleton@sydney.edu.au)

**What if I have a complaint or any concerns?**

The ethical aspects of this study have been approved by the Human Research Ethics Committee (HREC) of The University of Sydney HREC Approval No.: 2023/081 according to the *National Statement on Ethical Conduct in Human Research (2007).* If you are concerned about the way this study is being conducted or you wish to make a complaint to someone independent from the study, please contact the University: Human Ethics Manager [human.ethics@sydney.edu.au](mailto:human.ethics@sydney.edu.au) +61 2 8627 8176

***This information sheet is for you to keep.***


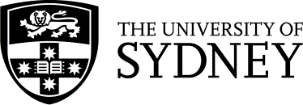
**EMPOWER-SMS GP RCT Participant Consent Form***

*REDCap text
__________________________________________

You are invited to take part in a research study (The Study). The study is run by a team at the University of Sydney, who are working with your General Practitioner (GP). The Study is testing if a new text message program called EMPOWER-SMS GP can help support people’s health and wellness alongside GP appointments after breast cancer treatment. The full information about the study can be found in the Participant Information Sheet: **[hyperlink to Participant Information Sheet]**
If you agree to take part, you will:

**1) Receive the 6-month EMPOWER-SMS GP program. You have a 50% chance of receiving the program this week or in 2-years.** EMPOWER-SMS GP was designed by breast cancer survivors, health professionals and researchers. Feedback from over 500 breast cancer survivors found that EMPOWER-SMS GP was easy-to-understand (99%), useful (91%), motivating for exercise and healthy eating (70%) and helped people feel supported (87%) between medical appointments. The short messages (1-2 sentences) are meant to support care from your GP and link you to helpful information or free health programs. But, this program does not replace advice from qualified healthcare professionals. You don’t need to reply and can read the messages at a good time for you. Please see the Participant Information Sheet for more details about the text messages.

**2) Fill in online surveys today, and again in 6-, 12-, 18-, and 24-months.** The surveys take between 5-10 minutes to fill in, depending on the month.

**3) Wear an activity watch for 7 days at the start of the study (next week) and again 6-months later.** This watch only collects information about your physical activity levels and sleep. It does not collect information about your location. People of all activity levels (beginner to advance) are encouraged to take part.

**Your Confirmation and Consent to participate in the study**

| In giving my consent, that I understand and have read the Participant Information Sheet and agree to take part in the Study as described. I confirm that I (am):   - Over 18 years of age and own a mobile phone - Completed active breast cancer treatment within the last 3 years (can still be taking endocrine/anti-hormone treatment) and not diagnosed with metastatic breast cancer - **Willing to wear an activity watch for 7 days** - Can read and understand English (at a grade 7 level) - Able to do all study activities (for example, you can eat vegetables and get active - you are not limited for medical reasons) - Not taking part in another text message study or clinical trial - In agreement that health data collected by my GP (such as medication list and medical history) can be auto extracted from their computer system for the purposes of this study only. I understand all data collected will be kept confidential and stored securely. - In agreement that researchers can send me text messages via Burst SMS. I understand that any text messages I send to or receive from the study mobile number will be stored on the Burst SMS platform according to their privacy policy and only used for the purposes of this study. | 🞎 Yes No  If no🡪 Reasons for not taking part (please specify):___ |
| --- | --- |
| **First Name** | Free text |
| **Surname** | Free text |

SUBMIT🡪

*Please note: After participants click submit, they will be directed to the REDCap baseline demographics survey.
