# Supplementary material for: Text message intervention delivered from Australian general practices to improve breast cancer survivors’ physical activity and cardiovascular risk factors: protocol for the EMPOWER-SMS-GP effectiveness implementation randomised controlled trial
Source: BMJ Open. 2024 Dec 11;14(12):e090984. doi: 10.1136/bmjopen-2024-090984 (PMC11647304; doi:10.1136/bmjopen-2024-090984)
Supplement: Supplementary file 3 [file bmjopen-2024-090984.reviewer_comments.pdf]

PEER REVIEW HISTORY

BMJ Open publishes all reviews undertaken for accepted manuscripts. Reviewers are asked to complete a checklist review form and are provided with free text boxes to elaborate on their assessment. These free text comments are reproduced below.

ARTICLE DETAILS

Title (Provisional)

A text message intervention delivered from Australian general practices to improve breast cancer survivors' physical activity and cardiovascular risk factors: protocol for the EMPOWER-SMS-GP effectiveness-implementation randomised controlled trial

Authors

Singleton, Anna C; Partridge, Stephanie R; Hyun, Karice K; Mitchell, Christine; Raeside, Rebecca; Hafiz, Nashid; Nickel, Brooke; Mizrahi, David; Todd, Allyson Ruth; McIntosh, Jennifer; Chan, Raymond J; Stuart, Kirsty E; Ee, Carolyn; Elder, Elisabeth; Redfern, Julie

This article was not externally reviewed at BMJ Open. Protocols that have been independently assessed prior to submission to BMJ Open are usually fast-tracked to publication on the grounds that further substantial changes will not be possible. This independent assessment will usually be external, independent review for both a substantial grant award from a non-commercial or government funder and ethics approval.
